# Variability in phase and amplitude of diurnal rhythms is related to variation of mood in bipolar and borderline personality disorder

**DOI:** 10.1038/s41598-018-19888-9

**Published:** 2018-01-26

**Authors:** O. Carr, K. E. A. Saunders, A. Tsanas, A. C. Bilderbeck, N. Palmius, J. R. Geddes, R. Foster, G. M. Goodwin, M. De Vos

**Affiliations:** 1 Department of Engineering Science, Institute of Biomedical Engineering, University of Oxford, Oxford, OX3 7DQ UK; 2 Department of Psychiatry, University of Oxford, Oxford, OX3 7JX UK; 3 Oxford Health NHS Foundation Trust, Warneford Hospital, Oxford, OX3 7JX UK; 4 Usher Institute of Population Health Sciences and Informatics, Medical School, University of Edinburgh, Edinburgh, EH16 4UX UK; 5 Oxford Centre for Industrial and Applied Mathematics, Mathematical Institute, University of Oxford, Oxford, OX2 6GG UK; 6 Sleep and Circadian Neuroscience Institute, Nuffield Department of Clinical Neurosciences, University of Oxford, Oxford, OX3 9DU UK

## Abstract

Variable mood is an important feature of psychiatric disorders. However, its measurement and relationship to objective measureas of physiology and behaviour have rarely been studied. Smart-phones facilitate continuous personalized prospective monitoring of subjective experience and behavioural and physiological signals can be measured through wearable devices. Such passive data streams allow novel estimates of diurnal variability. Phase and amplitude of diurnal rhythms were quantified using new techniques that fitted sinusoids to heart rate (HR) and acceleration signals. We investigated mood and diurnal variation for four days in 20 outpatients with bipolar disorder (BD), 14 with borderline personality disorder (BPD) and 20 healthy controls (HC) using a smart-phone app, portable electrocardiogram (ECG), and actigraphy. Variability in negative affect, positive affect, and irritability was elevated in patient groups compared with HC. The study demonstrated convincing associations between variability in subjective mood and objective variability in diurnal physiology. For BPD there was a pattern of positive correlations between mood variability and variation in activity, sleep and HR. The findings suggest BPD is linked more than currently believed with a disorder of diurnal rhythm; in both BPD and BD reducing the variability of sleep phase may be a way to reduce variability of subjective mood.

## Introduction

Bipolar disorder (BD) is associated with periods of elated and depressed mood interspersed by periods of relative stability or euthymia. Major mood episodes can last for weeks to months^1,2^. However, even when well, people with BD experience considerable inter-episode mood instability. Such instability is not unique to BD. It is also reported as a major problem by patients with borderline personality disorder (BPD) and is present in a surprising 14% of the general population in whom it appears to be a risk factor for a variety of negative psychiatric outcomes, including suicide^3^.

Mood instability is not well understood. One way to imagine its origin is to start from mechanisms that confer stability on biological systems. The best characterised example is perhaps the circadian or clock system present in all cells and particularly highly organized for regulation of brain function. Disruption of sleep is observed to be a prominent feature of psychiatric disorders in general and BD in particular^4,5^, which will operate through the homeostat, and the possible intrinsic variability in the diurnal rhythm of the clock. The effects of the clock are obvious in relation to jet lag, before the clock resets to local time after long haul travel. More subtle disruption of the clock’s diurnal rhythm has been studied infrequently in psychiatric patients. Thus, descriptions of sleep disturbance tend to be informal and do not distinguish between homeostat and clock mechanisms in its causation.

Mood instability in BD disorder has been widely shown. Increased mood variability in euthymic BD patients compared to healthy controls has been shown through retrospective questionnaires, indicating BD is not restricted to episodes of depression and mania but is also associated with mood instability between episodes^6^. More recent studies have used more continuous daily monitoring of mood in BD with smartphones, again finding increased mood variability in inter-episodic periods^7,8^. Patients with BPD are not as widely studied, however similar affective lability has been described in BPD^9^. Daily self-reported mood collected using a novel smartphone app reveals increased mood variability in both BD and BPD^10^. In addition, mood variability measures were able to discriminate between the three subject groups, suggesting mood varies differently across the disorders.

Stabilization of diurnal rhythms in BD is the objective of a number of current treatments^11^. Initial studies on BD measured diurnal disturbance through alterations to the sleep-wake cycle using simple estimates of sleep onset and offset from diaries and questionnaires^12^. More recently, actigraphy and melatonin levels have been employed as more objective measures^13–15^. Longer term recordings of actigraphy allow a wider range of diurnal measures to be calculated, for example the least active 5 hours of nocturnal activity (L5), most active 10 hour period or average daily activity^16^. These measures, in addition to quantifying the phase of 24 hours of activity through fitting a sinusoid, found BD participants to have less regular diurnal rhythms compared to controls^17,18^. There have been far fewer studies on diurnal disruption in BPD but a relatively recent review by Fleischer *et al*. described evidence of altered diurnal rhythms in BPD, along with considerable sleep disturbance^19^. Preliminary candidate gene studies have also suggested a link between circadian genes and susceptibility to BD^20^.

Variability in diurnal rhythm or sleep may contribute to mood symptoms in BD and BPD. Variability in diurnal function can be quantified as activity in sleep-wake cycle variability^21^. Episodes of altered mood are associated with significant changes in activity levels, making measures of activity a potential marker for mood variability^2^. In addition, subtle changes in temperature and autonomic nervous system (ANS) function also vary in a diurnal cycle. Broadly speaking, sympathetic nervous system activity is activating (e.g. increasing heart rate (HR)), whereas parasympathetic nervous system activity imposes resting states in the body (e.g. returning the HR to a normal level)^22^. If diurnal instability is related to mood instability, objective measures of variability in activity, sleep and HR should correlate with variability of subjective experience^1,17^.

BD and BPD are important conditions with high morbidity and premature mortality (from suicide and cardiovascular disease)^23^. Both are associated with variable mood, but BPD displays pervasive poor impulse control, instability of interpersonal relationship and disturbed self-image, with patients often expressing aggression in association with their emotional distress^24^. BPD and BD can be difficult to distinguish because of the overlapping features of mood instability and impulsivity, but mania is only seen in BD. BPD has been associated with disruptions to the sleep-wake cycle and irregular behavioural patterns^19^, along with disruptions in the ANS^25^.

Diagnosis and monitoring of both BD and BPD has largely relied on retrospective self-reporting of symptoms, which is inherently unreliable and prone to recall bias^26^. We had previously demonstrated that mood variability differed significantly between BD, BPD, and healthy control (HC) participants, when monitored over a longitudinal (*multiple months*) period^10,27^. Here, daily assessment of mood symptoms has been made using a compact clinical scale called Mood Zoom (MZ) delivered via a bespoke smart-phone app^28^. The responses to the MZ questionnaire have been shown to be highly correlated with the results of traditional questionnaires (QIDS^29^, GAD-7^30^, and EQ-5D) for mood, anxiety and quality of life^10^.

The aims of this study were to: (a) develop a method of quantifying diurnal rhythm regularity, (b) explore how diurnal variability of activity, sleep, HR and variability in mood are altered in BD, BPD and HC (c) investigate the links between diurnal variability and mood variability in BD and BPD participants.

## Data

The data was collected as part of the Automated Monitoring of Symptom Severity (AMoSS) study at the University of Oxford^28^. Participants gave written informed consent for a protocol approved by the NRES Committee East of England - Norfolk (13/EE/0288), all methods were carried out in accordance with the Code of Ethics of the World Medical Association (Declarations of Helsinki of 1975) for experiments involving humans. The AMoSS study collected behavioural data from participants^31^, in addition to self-reported mood scores using a smartphone app, as well as self-reported clinically validated questionnaires to determine psychiatric state^10,27^. Participants diagnosed with BD or BPD and healthy controls were recruited for the study through previous studies, local advertising and word-of-mouth. All the participants were screened by an experienced psychiatrist (KEAS) using the Structured Clinical Interview for DSM IV. They agreed initially for a three month period of recording, with the option of continuing participating in the study and providing mood recordings beyond the first three months. Data collection started in March 2014 and the data analysed here was collected up until February 2016, with 54 BD, 34 BPD and 53 HC participants recruited.

For one week during the study, the participants underwent ‘high intensity’ monitoring, during which multiple signals were recorded from a Proteus patch (www.proteus.com), stuck to the torso of participants. The patch was not removed during the whole ‘high intensity’ week and recorded HR and acceleration in three directions at one sample per minute.

Mood data was collected through the MZ smart-phone application, which prompted participants to provide their mood ten times evenly spaced throughout each day. The prompts typically started at 10am and finished at 8pm, thus on average mood characteristics were self-reported every hour. Participants rated six mood items in the smart-phone application: anxious, elated, sad, angry, irritable and energetic on a seven point Likert scale from zero, corresponding to ‘not at all’, to six, corresponding to ‘very much’. For further details on MZ see Tsanas *et al*.^10^.

The HR data obtained from the Proteus patch gave an average over five minute intervals; it did not permit measurement of conventional beat-to-beat heart rate variability. However, the data allowed for long term analysis of diurnal patterns in physiological and behavioural measures. The acceleration signals are recorded in vertical, horizontal and forward directions, with a sampling frequency of 0.017 Hz (once per minute). The reliability of the patches was variable so recordings from individual participant ranged from complete failure to eight days. Only participants who had four or more days of recording were included to allow a good representation of diurnal variability; the first four days of the recording were selected in order to standardise the analysis across all participants. The first day of ‘high intensity’ monitoring varied and we have not discriminated between weekdays and weekends.

Disposal of participants is shown in Table 1. Recordings were attempted on 113 participants (43 BD, 26 BPD and 44 HC) and satisfactory data analysed from 20 BD participants, 14 BPD participants and 20 HC. Of the 113 participants where recordings were attempted, 22 were discarded due to the devices failing to record any data, with the other 37 discarded as the recordings were shorter than four days.

**Table 1:** Numbers of participants recruited for the study and who had high intensity week recordings. Information for the participants who had data processed for analysis.

	Bipolar Disorder	Borderline Personality Disorder	Healthy Controls
Originally Recruited			
Participants	54	34	53
**High Intensity Recordings**			
Participants	43	26	44
**Processed Data**			
Participants	20	14	20
Gender	6 male; 14 female	2 male; 12 female	4 male; 16 female
Age (mean ± std)	41.0 ± 11.4	34.3 ± 10.2	43.4 ± 14.6
BMI (mean ± std)	26.1 ± 3.9	25.9 ± 5.1	24.6 ± 4.2
QIDS (mean ± std)	7.78 ± 5.50	12.35 ± 4.70	2.04 ± 1.53
Altman (mean ± std)	1.06 ± 1.21	2.69 ± 3.17	0.15 ± 0.67
Any psychotropic medication	18	10	0
Lithium	7	0	0
Anticonvulsant	11	1	0
Antipsychotic	11	3	0
Antidepressant	7	10	0
Hypnotics	1	0	0
Anxiolyticz	1	3	0

BD and BPD participants were taking a range of different medications (Table 1); BD: anticonvulsants, antipsychotics, lithium and antidepressants; BPD: mainly antidepressants. All correlations described below ignored medication. This is because there were no obvious differences in the pattern between variability of mood, variability of diurnal measures or correlations with and without medication when statistical tests were applied.

## Methods

### Mood Zoom Data

Principal component analysis was previously applied to the MZ questionnaire, with the first three components used to quantify the negative, positive and irritable moods of participants^10^. Variability of MZ scores were quantified using the standard deviation, the mean of the Teager-Kaiser energy operator (TKEO), entropy, and root mean squared successive differences (RMSSD) of each mood score. The MZ variability measures are defined as:

1 $$std=\sqrt{\frac{1}{N}\sum _{i=1}^{N}{({x}_{i}-{\mu }_{x})}^{2}}$$

2 $$TKEO=\frac{1}{N}\sum _{i=2}^{N-1}({x}_{i}^{2}-{x}_{i-1}{x}_{i+1})$$

3 $$entropy=\sum _{i=1}^{N}p({x}_{i})\mathrm{log}(p({x}_{i}))$$

4 $$RMSSD=\sqrt{\frac{1}{N}{\sum _{i=1}^{N-1}({x}_{i+1}-{x}_{i})}^{2}}$$

where *N* is the total number of samples in the signal (number of MZ entries), *x*_*i*_ is the *i*^*th*^ value of the signal (*i*^*th*^ MZ entry) and *μ*_*x*_ is the mean of the signal (mean MZ entry).

The variability measures were calculated on the principal components of MZ for the four days corresponding to the HR and actigraphy recordings. The approximate sampling frequency is one entry per hour, however there will be a nightly gap for each 24 hour period where no MZ entries are recorded.

### Extracting Diurnal Rhythms

**Figure 1:**
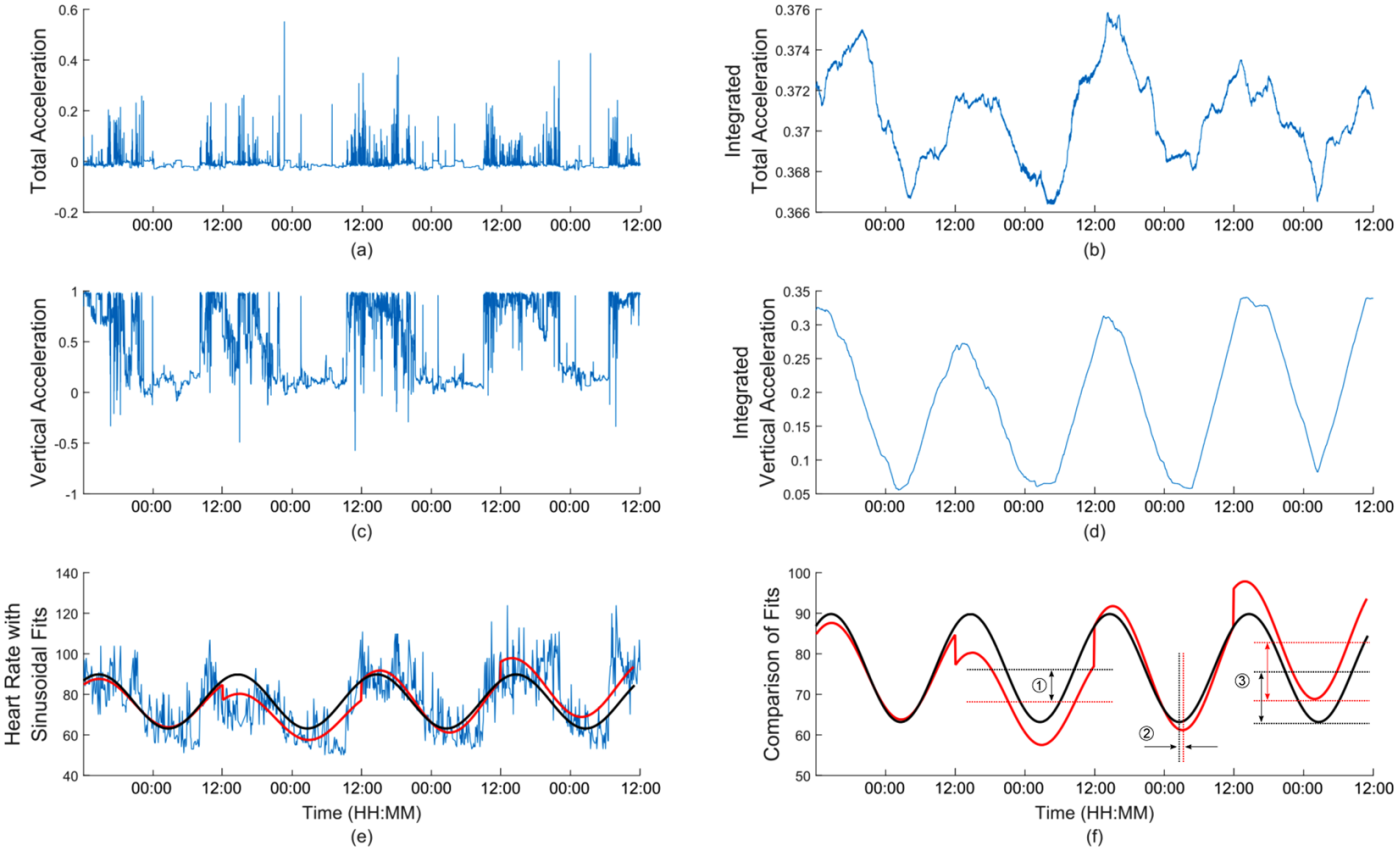
An example of data recorded for the Proteus patch for one participant. (**a**) total acceleration, (**b**) the integrated total acceleration, (**c**) vertical acceleration, and (**d**) the integrated vertical acceleration. (**e**) the HR with a sinusoid fit to each 24 hour period in red and to the total data in black. (**f**) the comparison between daily and total sinusoids with difference between midline estimating statistic of rhythms (MESORs) ①, phases ② and amplitudes ③ shown.

HR data was pre-processed to remove artefacts in the signal. The value of HR at each recorded instance was compared to the value recorded at the preceding time instance: if the value was over 150% or under 50% of the previous value the value at the current time was set to the mean of the preceding and succeeding values. Artefacts were typically only one sample long and imputing values based on an average over a larger period had no impact on results, therefore it was decided to use the simple approach of averaging previous and succeeding values. A mean of 1% ± 1.2% of the data was adjusted due to artefacts for each participant. An indicative example of the recorded data is presented in Fig. 1. The HR signals exhibited a natural sinusoidal behaviour (Fig. 1e). The acceleration signals did not show this pattern as clearly, therefore the signals were integrated to emphasise this sinusoidal behaviour (see Fig. 1b and d). The integration was performed by centring the signals on zero by subtracting the means, and then integrating an overlapping window of six hours which is shifted by one data point along the entire signal.

Quantification of diurnal rhythms allowed for the analysis of behavioural and physiological regularity within participants or subject groups. A number of previous studies on diurnal rhythms of HR have performed fitting of a sine wave to a 24 hour signal by quantifying the phase, average value and amplitude, known as the Cosinor method^32–34^. Here, this analysis has been extended to longer term signals over a number of days through fitting of sine waves to both the entire signal and to each 24 hour section of the signal for integrated total acceleration, integrated vertical acceleration and HR (see Fig. 1b,d and e).

The diurnal analysis was performed on the HR signal, in addition to the integrated total acceleration signal (a measure of total activity) and the integrated vertical acceleration signal (a measure of sleep or rest-activity, due to changing reference angle of the wearer). We fitted directly to the raw HR signal from midday to midday in order to emphasise the night time behaviour, as shown in red. The sinusoids were fit with a fixed period of 24 hours and the amplitude and phase were optimised to give a least squares fit. Secondly, a sinusoidal fit was carried out on the entire signal to provide an average diurnal pattern for the length of the signal, with an identical fitting method as the daily fits, shown in black. The same technique of fitting sinusoids was applied to the integrated total acceleration signal, to give a diurnal measure of activity and also to the integrated vertical acceleration; this gave a diurnal measure of lying down, used as a proxy for measuring sleep or rest-activity levels.

### Diurnal Parameters

Diurnal features were calculated by comparing the daily sinusoid fits of HR, total acceleration and vertical acceleration to the total sinusoid fits to the same data. The similarity between the daily and total fits indicates the degree of diurnal regularity during the period of measurement.

**Figure 2:**
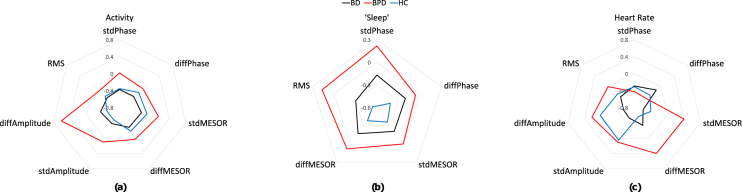
Standardised median diurnal feature values across the three subject groups, separated into activity (**a**), sleep (**b**) and HR (**c**) features. stdPhase, stdMESOR and stdAmplitude denote the standard deviation of the differences between the daily and total sinusoid fit. diffPhase, diffMESOR and diffAmplitude denote the standard deviation of successive differences in the daily sinusoid fits. RMS is the root mean square error between the daily and total sinusoid fits. Measures were standardised by subtracting the mean and dividing by the standard deviation of each feature for the groups combined. Standardisation was only performed to visually compare measures in this figure.

**Figure 3:**
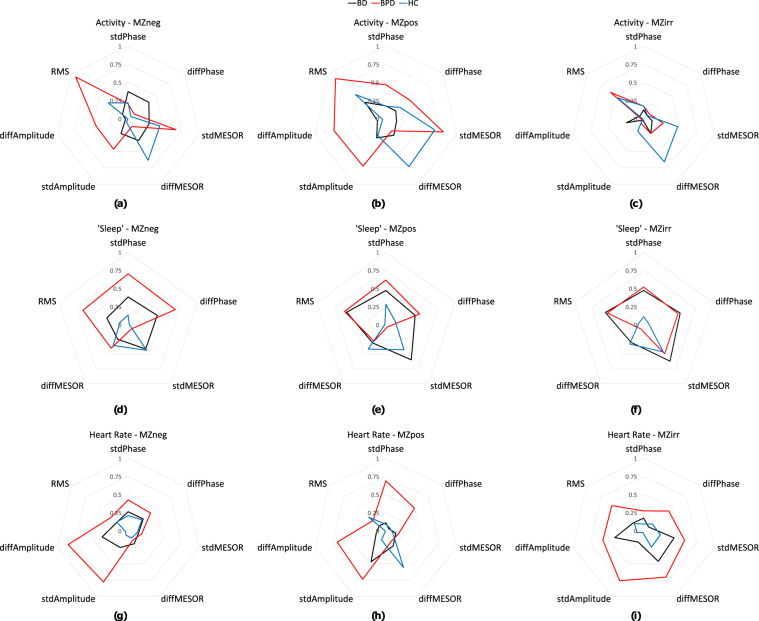
Absolute correlation coefficients between diurnal variability measures and standard deviation of MZ measures to show how correlations vary across subject groups. Each spider plot represents a pair of diurnal HR, acceleration or vertical acceleration with negative MZ, positive MZ or irritable MZ measures. stdPhase, stdMESOR and stdAmplitude denote the standard deviation of the differences between the daily and total sinusoid fit. diffPhase, diffMESOR and diffAmplitude denote the standard deviation of successive differences in the daily sinusoid fits. RMS is the root mean square error between the daily and total sinusoid fits.

Differences between the fits were measured through differences in the midline estimating statistic of rhythm (MESOR) (Fig. 1f, ①), or average, the phase (Fig. 1f, ②) and the amplitude (the difference between a and b) (Fig. 1f, ③); the timings of the minimum values of the daily sinusoidal fits are found (*Td*), as well as the amplitudes (*Ad*) and MESORs (*Md*), in addition to the timings of the minimum values for the total (or weekly) sinusoidal fit (*Tw*) and the corresponding amplitudes (*Aw*) and MESORs (*Mw*). Details of the calculations are given as Supplementary Material.

These new measures quantify regularity of diurnal rhythms over longitudinal recordings. They encapsulate how the timings (phase) of the rhythms are shifting along with how both the average (MESOR) and range (amplitude) of recordings differ from day to day, as well as giving an overall measure of diurnal regularity through the RMS error between the daily and total sinusoids. The standard measures reported in the literature are based on actigraphy measures defined almost two decades ago: these include most active and least active periods and transitions between rest and activity^16^. A Cosinor method has also been used which quantifies phase, amplitude and MESOR, and can be generalised to other recordings (such as heart rate^34^). However, the new measures which find the difference from an average or total Cosinor fit for each day allow for better measures of regularity on an individual participant level.

### Statistical Tests

Our analysis plan tested the hypothesis that variability of diurnal rhythms would be associated with variation in mood. We investigated differences between the groups through diurnal features extracted from HR, integrated total acceleration and integrated vertical acceleration using the Wilcoxon rank sum test. To compute the correlation coefficients between diurnal features and MZ features, outliers were removed by calculating the Cook distance of each point: if the Cook distance of a point was greater than three times the mean Cook distance the point was removed as an outlier. An average of 1.6 ± 0.4 participants were removed as outliers for the calculation of the correlation coefficient for each pair of mood and diurnal measures. After outliers had been removed, Pearson correlation coefficients along with corresponding statistical significance (p-values) for each pair of diurnal feature and MZ feature were calculated. Correction for multiple statistical hypothesis tests was performed using the positive false discovery rate (significance at the 5% level), as defined by Storey, 2002^35^. The positive false discovery rate aims to reduce the number of type I errors (incorrectly rejecting the null hypothesis). Here, the false discovery rate correction was separately applied to the statistical tests for: comparison of mood variability, diurnal variability and correlations.

### Data availability

The datasets analysed during the study are available from the corresponding author on reasonable request.

## Results

### Mood Zoom variability

There were very large differences in all measures of daily MZ variability in negative mood, with BPD participants more variable than BD, who were more variable than HC (almost all differences are statistically significant, Table 2). A similar pattern was observed for daily measures of irritability and, to a lesser degree, positive mood. All pairwise comparisons were found to be statistically significant, with the exception of comparisons of positive MZ variability between BD and HC participants. Principal component weightings are described in more detail in Tsanas *et al*.^10^.

**Table 2:** Comparing variability of all MZ recordings during the high intensity week across the three groups, and statistical significance pairwise comparisons across the three groups (BD, BPD, HC) using the Wilcoxon statistical hypothesis test and false discovery rate.

	BD (median ± iqr)	BPD (median ± iqr)	HC (median ± iqr)	BD vs BPD (FDR)	BD vs HC (FDR)	BPD vs HC (FDR)
MZnegstd	0.99 ± 0.85	1.71 ± 1.11	0.35 ± 0.47	**1.57E-03**	**2.31E-02**	**1.79E-04**
MZnegTKEO	0.79 ± 0.88	1.94 ± 4.86	0.12 ± 0.43	**1.37E-02**	**4.78E-02**	**6.62E-04**
MZnegRMSSD	1.07 ± 0.64	1.69 ± 1.46	0.42 ± 0.62	**2.02E-03**	**2.23E-02**	**2.03E-04**
MZposentropy	−6.67 ± 122.65	6.67 ± 3.91	−118.28 ± 414.04	**4.34E-03**	**7.64E-02**	**7.76E-04**
MZposstd	0.91 ± 0.70	1.42 ± 0.56	0.62 ± 0.52	**1.21E-03**	6.13E-01	**7.76E-04**
MZposTKEO	0.39 ± 0.77	1.40 ± 1.04	0.23 ± 0.54	**2.02E-03**	9.25E-01	**4.34E-03**
MZposRMSSD	0.91 ± 0.52	1.38 ± 0.54	0.66 ± 0.64	**1.87E-03**	7.35E-01	**1.87E-03**
MZposentropy	−9.71 ± 134.27	3.83 ± 8.86	−65.57 ± 143.14	**2.18E-03**	**4.14E-01**	**1.49E-03**
MZirrstd	0.56 ± 0.43	1.01 ± 0.49	0.33 ± 0.46	**1.87E-03**	**2.39E-02**	**2.98E-04**
MZirrTKEO	0.24 ± 0.31	0.81 ± 0.69	0.09 ± 0.34	**1.25E-03**	**5.43E-02**	**4.78E-04**
MZirrRMSSD	0.71 ± 0.44	1.15 ± 0.41	0.40 ± 0.59	**2.02E-03**	**4.34E-02**	**6.62E-04**
MZirrentropy	−23.90 ± 92.84	−3.50 ± 13.93	−188.99 ± 547.87	**2.64E-03**	**5.27E-02**	**7.76E-04**

Statistically significant differences at the FDR = 0.05 level appear in bold. “MZneg” denotes the negative principal component of MZ, “MZpos” denotes the positive principal component of MZ, and “MZirr” the irritability principal component of MZ computed using the PCA loadings^10^.

### Diurnal variability: Heart rate, activity and sleep

The patterns of diurnal variability of participant groups can be best appreciated in spider plots of the medians of the standardised diurnal features for activity, sleep, and HR (Fig. 2). Figure 2 shows standardised measures, obtained by subtracting the mean and dividing by the standard deviation of each feature for the groups combined. Standardisation was only performed to compare metrics visually in Fig. 2. On inspection variability of activity measures was increased in the BPD; BD and HC were not distinguishable. For sleep, variability was greater in BPD participants for phase and amplitude measures; BD was also variable, but intermediate between BPD and HC. For HR, BPD group showed an increase in the MESOR compared with HC; BD and HC were similar (Fig. 2a). No standardisation was performed for statistical analysis and the observed differences represent trends which did not survive correction for multiple comparison (Table 3).

**Table 3:** Comparing variability of diurnal features of HR, activity and sleep across the three groups, and statistical significance pairwise comparisons across the three groups (BD, BPD, HC) using the Wilcoxon statistical hypothesis test.

Diurnal Features	BD (median ± iqr)	BPD (median ± iqr)	HC (median ± iqr)
**Total Acceleration**			
stdPhase	1.48 ± 0.05	2.18 ± 0.09	1.52 ± 0.04
stdMESOR	0.0007 ± 0.0005	0.0009 ± 0.0005	0.0008 ± 0.0005
stdAmplitude	0.0006 ± 0.0004	0.0009 ± 0.0007	0.0006 ± 0.0006
diffPhase	2.29 ± 0.08	3.15 ± 0.16	2.75 ± 0.08
diffMESOR	0.0010 ± 0.0006	0.0012 ± 0.0006	0.0010 ± 0.0008
diffAmplitude	0.0009 ± 0.0007	0.0018 ± 0.0014	0.0008 ± 0.0010
RMS	0.000001 ± 0.000001	0.000001 ± 0.000001	0.000001 ± 0.000001
‘**Sleep**’			
stdPhase	0.93 ± 0.03	1.17 ± 0.05	0.67 ± 0.02
stdAmplitude	0.017 ± 0.016	0.019 ± 0.017	0.015 ± 0.010
diffPhase	1.298 ± 0.048	1.462 ± 0.104	1.057 ± 0.023
diffAmplitude	0.03 ± 0.02	0.03 ± 0.03	0.02 ± 0.02
RMS	0.001 ± 0.001	0.001 ± 0.001	0.001 ± 0.001
**Heart Rate**			
stdPhase	1.715 ± 0.074	1.467 ± 0.080	1.681 ± 0.104
stdMESOR	1.768 ± 1.756	3.196 ± 1.837	2.036 ± 1.422
stdAmplitude	2.03 ± 1.94	2.80 ± 2.06	2.74 ± 1.33
diffPhase	2.69 ± 0.10	1.94 ± 0.09	2.24 ± 0.11
diffMESOR	2.86 ± 3.71	4.81 ± 4.66	2.25 ± 2.46
diffAmplitude	2.80 ± 2.16	4.60 ± 2.57	4.13 ± 2.07
RMS	11.24 ± 9.81	16.71 ± 15.64	12.64 ± 12.81

No statistically significant differences were found after FDR correction.

### Correlations between variation in mood and variation in diurnal measures

Correlations were calculated between the seven diurnal HR features, the seven diurnal activity features and the five diurnal sleep features and the standard deviation of MZ components. Statistically significant positive correlations were found in 14 of these pairs (after correcting for false discovery rate), shown in Table 4.

**Table 4:** Correlation coefficients between diurnal features and MZ features for BD, BPD and HC participants.

Diurnal Features	BD			BPD			HC		
	Correlation Coefficient			Correlation Coefficient			Correlation Coefficient		
	MZneg	MZpos	MZirr	MZneg	MZpos	MZirr	MZneg	MZpos	MZirr
**Total Acceleration**									
stdPhase	−0.374	−0.178	−0.122	0.206	0.470	0.174	0.219	0.174	0.176
stdMESOR	0.298	0.151	0.120	0.676	0.812**	0.276	0.448	0.691*	0.481
stdAmplitude	0.226	−0.292	0.020	0.465	0.724*	0.074	−0.052	0.258	−0.190
diffPhase	−0.365	−0.174	−0.065	0.105	0.417	−0.104	0.049	0.251	−0.069
diffMESOR	0.332	0.252	0.225	−0.119	0.185	0.222	0.635	0.731*	0.658
diffAmplitude	−0.037	−0.120	−0.241	0.452	0.733*	0.029	0.004	0.046	−0.012
RMS	0.100	−0.363	−0.065	0.923***	0.887**	0.583	0.350	0.535	0.464
**‘Sleep’**									
stdPhase	0.384	0.473	0.474	0.703*	0.617	0.521	−0.134	0.282	−0.112
stdAmplitude	0.411	0.595	0.618	−0.071	−0.035	0.492	−0.433	−0.423	−0.462
diffPhase	0.422	0.424	0.529	0.683	0.487	0.498	0.017	0.139	0.07
diffAmplitude	0.245	0.308	0.297	−0.397	−0.277	−0.068	−0.351	−0.413	−0.326
RMS	0.309	0.567	0.555	0.655	0.599	0.525	−0.118	0.015	0.071
**Heart Rate**									
stdPhase	−0.265	0.112	−0.177	0.427	0.690	0.276	−0.211	−0.092	−0.094
stdMESOR	−0.145	0.141	−0.430	0.190	0.177	0.579	−0.125	0.096	−0.235
stdAmplitude	−0.253	0.471	−0.171	0.781**	0.740*	0.761**	0.066	−0.139	−0.026
diffPhase	−0.265	0.051	−0.087	0.395	0.503	0.441	−0.244	−0.068	−0.153
diffMESOR	−0.195	0.229	−0.465	0.143	0.163	0.706*	0.108	0.559	0.248
diffAmplitude	−0.367	0.136	−0.407	0.848**	0.687*	0.576	−0.038	−0.006	−0.092
RMS	−0.195	0.115	−0.180	0.301	0.228	0.561	−0.198	0.303	−0.165

Pearson correlation coefficients, with statistically significant correlations appearing in bold. ^*^Represents the FDR = 0.05 level, ^**^The FDR = 0.01 level and ^***^The FDR <0.001 level. For diurnal features, stdPhase, stdMESOR and stdAmplitude denote standard deviation of the difference between daily and total sinusoids measures. diff indicates the feature is the standard deviation of successive differences.

For total acceleration, BD showed no consistent correlations with mood, and no trend even for small positive correlations. This contrasted with the BPD and HC groups where all mood dimensions were highly positively correlated with variation in the stdMESOR, a measure of variability of average daily activity levels. BPD showed equally strong effect for RMS, a measure of overall variability of activity levels, but not diffMESOR, a measure of variability of day to day variability in average activity levels. HC showed a strong effect for diffMESOR and trended positive for RMS.

For the sleep measure, negative mood variability was highly correlated with stdPhase, a measure of variability of sleep timings, in BPD. High positive correlations were found between variability of all moon dimensions and variability of sleep phases in BPD and similarly in BD with slightly smaller correlations. These correlations were not seen in HC participants.

For the HR measure in BPD, variation in signal amplitude was strongly positively correlated with measures of emotion. Neither the BD or HC groups showed even trend positive effects.

The absolute correlation coefficients for the groups can be appreciated in Fig. 3. For example, Fig. 3i shows the correlation coefficients between each of the diurnal HR features and the standard deviation of irritable mood, with BPD participants having large correlation coefficients, thus having a larger radius. The HC show little correlation, with a small radius close to zero at the centre and the BD participants have small absolute correlations, thus a small radius. Another example in Fig. 3f shows BD and BPD participants having larger correlations between sleep and irritable mood variability through a larger radius, whereas the HC show few correlations.

For BPD participants what is notable is the generally larger correlation coefficients compared to the other groups for most correlations, but especially in the case of irritability and HR. For BD participants, vertical acceleration (sleep) showed clearly greater correlations with MZ measures than the HC group (and comparable with BPD).

For HC participants, activity showed relatively large correlation coefficients with MZ measures comparable with BPD and greater than BD.

## Discussion

This exploratory study demonstrated convincing associations between variability in subjective mood and variability in measures of diurnal physiology for BPD. The differences between patient groups and healthy controls are of considerable interest. The BPD group showed greater variation in both mood and in diurnal physiology, however measured. By contrast, BD showed intermediate variation in mood (less than BPD, more than HC) and increased variability only in diurnal measures of sleep (less than BPD, more than HC) and not total activity or HR. Direct associations between variation in subjective mood and objective measures of diurnal variability were also demonstrated. Mood dimensions in both BPD and BD appeared more sensitive to variation in the phase and depth of sleep than HC. In addition, BPD showed clear evidence of an association between variability of HR and variation in irritability, a key symptom of the disorder. Mood variability in BD did not show this relationship. This may suggest that BPD, but not BD, should be thought of as a disorder linked with disorder of diurnal rhythm, although both appear to be influenced by variation in sleep.

We had already observed the distinctive differences in the variability of subjective mood experienced by BPD, BD and HC groups^10^. BPD was associated with high daily and, particularly, *within* day variation in negative, positive and irritable mood dimensions compared with HC. BD variability was intermediate between BPD and HC. Recording the mood items up to ten times per day strongly discriminated the BPD group and also confirmed the clinical impression that mood is both more variable and at a higher frequency in BPD patients. High intensity monitoring can reduce the sampling interval required to acquire mood data in the future. The variation in the phase, average levels and amplitude of diurnal rhythms in BPD is a new observation. BPD was associated with the highest levels of variability in almost all the physiological measures as a trend and met conventional levels of significance for a number of individual measures versus BD and HC. In contrast BD was not generally more variable than HC on the measures of activity and HR; there were trend effects for sleep. However, overall, variation in the physiological measures was less than the observed subjective variability.

We have previously suggested that mood instability is an important feature of the phenotype of both BD and BPD^10^. Despite the apparently less discriminating difference in the variability of diurnal signals, there appeared to be an important correlation between them. These correlations were most striking for both the BPD group in relation to activity and HR measures (Table 4). The present study did not determine the direction of effect, so we cannot say that variation in diurnal cycles drives mood variability. However, in the case of BD, this is the direction of effect suggested by observation of mood change in response to more obvious sleep disruption lasting many days. It would support the emphasis on sleep regularity on a day to day basis enshrined in the social rhythm metric^36^ and other behavioural approaches to mood stabilization in BD^37^. More detailed measures of mood could be an important read out of the impact of effective treatment.

In relation to BPD, the observation that mood measures, especially irritability, are related to measures of sleep, activity and HR variability appears to be new. Previous measures of sleep in BPD have emphasized poor sleep quality and in some studies delayed sleep timing, but not a direct effect of variability per se. Again sleep regularity may be as important an objective for psychosocial interventions for BPD as for BD. This is not reflected in current practice^38^.

Variability in sleep was derived from the vertical component of the accelerometer worn on the chests of the participants. It is important that this signal was not the same as total acceleration. In fact, variability in total acceleration was not highly correlated with subjective parameters in the way that the vertical component was. However, it is of course not a measure of sleep per se and will include time lying down but not sleeping. The variability in the sleep cycle could nevertheless be driven either by variability in a hypothetical central clock or in the sleep homeostat.

Finally, a substantial number of patients were receiving medication. This appeared to have no average effect within or across groups either on variability or the correlations between objective measures and subjective measures through the statistical tests performed between participants with and without medication. On the face of it, a drug like lithium might be expected to reduce variability, but an observational sample is clearly not the optimal group in which to demonstrate such an effect.

The traditional measure of heart rate variability (HRV) derived from an ECG, is the most common analysis method used in the study of physiology in BD and other psychiatric disorders, with links to emotion^39^. While altered HRV has been reported in BD^40,41^, alterations to HRV in BPD are not as well defined^42,43^. Furthermore, in previous studies HRV measures were recorded on very controlled five minute sections of ECG, based on timings between every individual heart beats, where the participants were known to be at rest^40,41,44,45^. In contrast, the results presented in this study are over much longer periods when the participants are behaving as normal, therefore recording very different aspects of HR, representative of the spontaneous state and related to diurnal changes in HR.

The increased variability in diurnal measures of HR showed striking correlations with mood in BPD and much less so in BD and HC. Again, the direction of effect is not established by our observations, but it cannot be simply driven by activity or sleep. Instead it may support the idea that BPD really does show greater variability in the function of the central diurnal clock. It is the first time that diurnal irregularity has been shown to correlate with one of the core features of the BPD phenotype, mood instability. The absence of either greater variability in HR measures or a strong relationship with mood suggests that BD is much less likely to be well characterized by central instability of clock function, which we did not predict^1,46,47^.

This was an exploratory study, therefore we had no detailed hypotheses how mood variability might be linked to iteams of physiology, although, as previously explained, some link seemed plausible. A relatively large number of correlations were calculated between mood and diurnal variability, and strong correlations were only found between certain measures of diurnal variability and mood variability (Table 4). Moreover, the majority of correlations were found for the BPD group who had more variable mood than either BD or HC. Whether this is a result of stronger coupling between physiology and mood will require further work. However, the variability of diurnal measures in BPD is less different (from BD and HC) than the mood variability, so it may be.

Long term naturalistic monitoring with inconvenient equipment poses real challenges and we collected fewer complete and usable sets of data than planned because the available equipment proved less than ideal. Study of diurnal rhythms over longer periods could improve the findings reported here, as four days of data is a relatively short period and single abnormal days have large effects on fitting the data. The days of the week were not taken into account either, so participants may have been recorded over four working days, which might be relatively regular, or they may be recorded for two working days and two weekend days, which we would expect to be less regular. This added variability may have helped the correlation analysis and will have tended to amplify effects in BPD who had the highest emotional variability.

We studied the participants in a relatively stable state as outpatients. It would be interesting, if difficult, to study severe mania and depression for the BD group. However, inter-episode states in BD or BPD are ‘at risk’ states and the mood/diurnal link is potentially important. Most interesting of all would be the transition from well to ill, but this presents a narrow observational window to a relatively rare event and so difficult to capture within the time frame of a prospective study.

Diurnal analysis of other measures like temperature, metabolic or inflammatory biomarkers may provide further insight into the links between diurnal function and mood, which could be closer to the brain mechanisms in BD or BPD.

The ability to detect a correlate of negative or unstable moods from objective measures, such as acceleration or HR, improves or complements the current methods of subjective report. With the right equipment, such measures could be largely frictionless. More continuous monitoring of participants suffering from mental disorders may lead to prediction of deteriorating mood states based on changes in diurnal patterns of objective markers, therefore allowing for preventative action to be initiated as part of a clinical management plan. Improved methods for collecting diurnal information over longer periods of time are required for confirmation and interpretation of diurnal signals under a wider range of states of illness.

The present study has established for the first time that variability in diurnal function is associated with variability of mood in BPD. It suggests that the associations are present for HR, total activity and the activity related to sleep. Any association between subjective experience and objective measures is unlikely to be simple or unidirectional. However, the results suggest that variable diurnal function is more characteristic of the BPD than the BD phenotype and so treatment efforts might be explored which would regularize circadian function as a way to regulate abnormal mood in BPD. New ideas are required to move this field forward and develop mental health care. Improved regularity of sleep may be an important potential target for therapy in both BD and BPD.

## Electronic supplementary material

Supplementary Information
