# Supplementary material for: Variability in phase and amplitude of diurnal rhythms is related to variation of mood in bipolar and borderline personality disorder
Source: Sci Rep. 2018 Jan 26;8:1649. doi: 10.1038/s41598-018-19888-9 (PMC5786095; doi:10.1038/s41598-018-19888-9)
Supplement: Supplementary file 1 — Supplementary Information [file 41598_2018_19888_MOESM1_ESM.pdf]

Variability in phase and amplitude of diurnal rhythms is related to variation of mood in bipolar and borderline personality disorder

O. Carr[1,*], K. E. A. Saunders[2,3], A. Tsanas[4,5], A. C. Bilderbeck[2], N. Palmius[1], J. R. Geddes[2,3], R. Foster[6], G. M. Goodwin[2,3,6], and M. De Vos[1,6]

[1]Institute of Biomedical Engineering, Department of Engineering Science, University of Oxford, Oxford, OX3 7DQ, UK
[2]Department of Psychiatry, University of Oxford, Oxford, OX3 7JX, UK
[3]Oxford Health NHS Foundation Trust, Warneford Hospital, Oxford, OX3 7JX, UK
[4]Usher Institute of Population Health Sciences and Informatics, Medical School, University of Edinburgh, Edinburgh, EH16 4UX, UK
[5]Oxford Centre for Industrial and Applied Mathematics, Mathematical Institute, University of Oxford, Oxford, OX2 6GG, UK
[6]Sleep and Circadian Neuroscience Institute, Nuffield Department of Clinical Neurosciences, University of Oxford, Oxford, OX3 9DU, UK
[*]oliver.carr@eng.ox.ac.uk

Supplementary Materials

Method for calculating variability parameters

The mean of the difference in timings between the daily and total minimums were calculated as:

$$\mu_T = \frac{1}{D}\sum_{i=1}^{D}(Td_i - Tw_i) \tag{1}$$

where D is the total number of days. The standard deviation of the difference in timings between the daily and total minimums is defined as:

$$\sigma_T = \sqrt{\frac{1}{D}\sum_{i=1}^{D}\left((Td_i - Tw_i) - \mu_T\right)^2} \tag{2}$$

Similarly, the mean (μ_A and μ_M) and standard deviation (σ_A and σ_A) of the difference between the amplitudes and MESORs of the daily and total sinusoids can be calculated from Ad and Aw and Md and Mw respectively. The residual sum of squares between the daily and total sinusoids is calculated as a total measure of regularity for the period of recording:

$$RSS = \sum_{i=1}^{N}\left(SIN_i^{day} - SIN_i^{week}\right)^2 \tag{3}$$

where SIN represents the fitted sinusoids.

In addition to regularity measures coming from the comparison of the daily sinusoids to the weekly sinusoids, means and standard deviations of successive differences of the daily sinusoids were found. The mean was found for the successive

differences of timings of the minimum values:

$$\mu DIF_T = \frac{1}{(D-1)} \sum_{i=1}^{D-1} (Td_{i+1} - Td_i) \tag{4}$$

with the standard deviation defined as:

$$\sigma DIF_T = \sqrt{\frac{1}{(D-1)} \sum_{i=1}^{D-1} \Big((Td_{i+1} - Td_i) - \mu DIF_T \Big)^2} \tag{5}$$

All these features were calculated for HR and acceleration data to give measures of the range of: HR and activity levels through the amplitude, the average HR and activity levels through the MESOR and the timings of the maximum or minimum HR and activity levels through the phase. As the vertical acceleration is a measure of sleep, the timings of the sleep can be indirectly measured through the phase and the amount of sleep or rest-activity, can be measured through the MESOR.
